# Δ-Bis[(*S*)-2-(4-isopropyl-4,5-di­hydro­oxazol-2-yl)phenolato-κ^2^*N*,*O*^1^](1,10-phenanthroline-κ^2^*N*,*N*′)ruthenium(III) hexa­fluorido­phosphate

**DOI:** 10.1107/S2414314624008939

**Published:** 2024-09-17

**Authors:** Monsuru T. Kelani, Alfred Muller, Koop Lammertsma

**Affiliations:** a Department of Chemical Sciences, University of Johannesburg, Auckland Park, 2006, Johannesburg, South Africa; Vienna University of Technology, Austria

**Keywords:** crystal structure, absolute configuration, ruthenium, chiral-at-metal complex

## Abstract

The title compound, [Ru(C_12_H_14_NO_2_)_2_(C_12_H_8_N_2_)]PF_6_ crystallizes in the tetra­gonal Sohnke space group *P*4_1_2_1_2. The two bidentate chiral salicyloxazoline ligands and the phenanthroline co-ligand coordinate to the central Ru^III^ atom through N,O and N,N atom pairs to form bite angles of 89.76 (15) and 79.0 (2)°, respectively. The octa­hedral coordination of the bidentate ligands leads to a propeller-like shape, which induces metal-centered chirality onto the complex, with a right-handed (Δ) absolute configuration [the Flack parameter value is −0.003 (14)]. Both the complex cation and the disordered PF_6_^−^ counter-anion are located on twofold rotation axes. Apart from Coulombic forces, the crystal cohesion is ensured by non-classical C—H⋯O and C—H⋯F inter­actions.

## Abstract 2

The title compound is an example for a chiral-at-metal complex, with the Ru^III^ atom having an octa­hedral coordination environment by three bidentate ligands.

## Graphical abstract

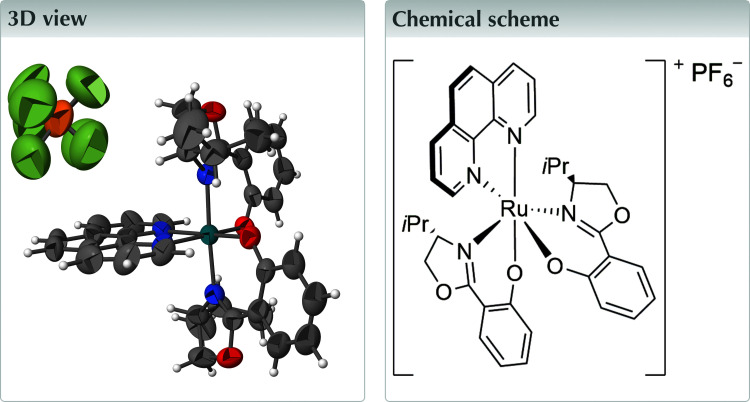

## Structure description

The syntheses of optically pure metal complexes are usually costly and sophisticated, especially with the use of traditional methods for the resolution of racemic mixtures. A straightforward alternative strategy, therefore, requires the coordination of pure chiral auxiliary ligands tailored for the selective synthesis of diastereomers, which are easily converted to the corresponding enanti­omerically pure complexes (Knof & von Zelewsky, 1999 ▸). Hayoz and co-workers were the first to report the diastereoselective synthesis of optically pure ruthenium polypyridyl complexes in the quest for generating compounds with metal-centered chirality, so-called chiral-at-metal complexes (Hayoz *et al.*, 1993 ▸). Such metal-centered chirality refers to the type of chirality induced at a central metal atom as a result of an helical octa­hedral coordination around a metal in bis-chelate or tris-chelate systems. In this context, optically pure salicyloxazoline is often used as an auxiliary ligand to implement and control the absolute configuration at central metal atoms during ligand exchange. In this case, the absolute configurations at the central metal could either be right-handed or left-handed twist systems, which are symbolized by Δ and Λ stereochemical descriptors, respectively (Gong *et al.*, 2010 ▸). The salicyloxazoline ligand is often used in this manner because of its reversible coordination upon acid protonation of its phenolate group while leaving the stereochemistry of the metal complex intact (Gong *et al.*, 2009 ▸, 2010 ▸, 2013 ▸).

**Figure 1:**
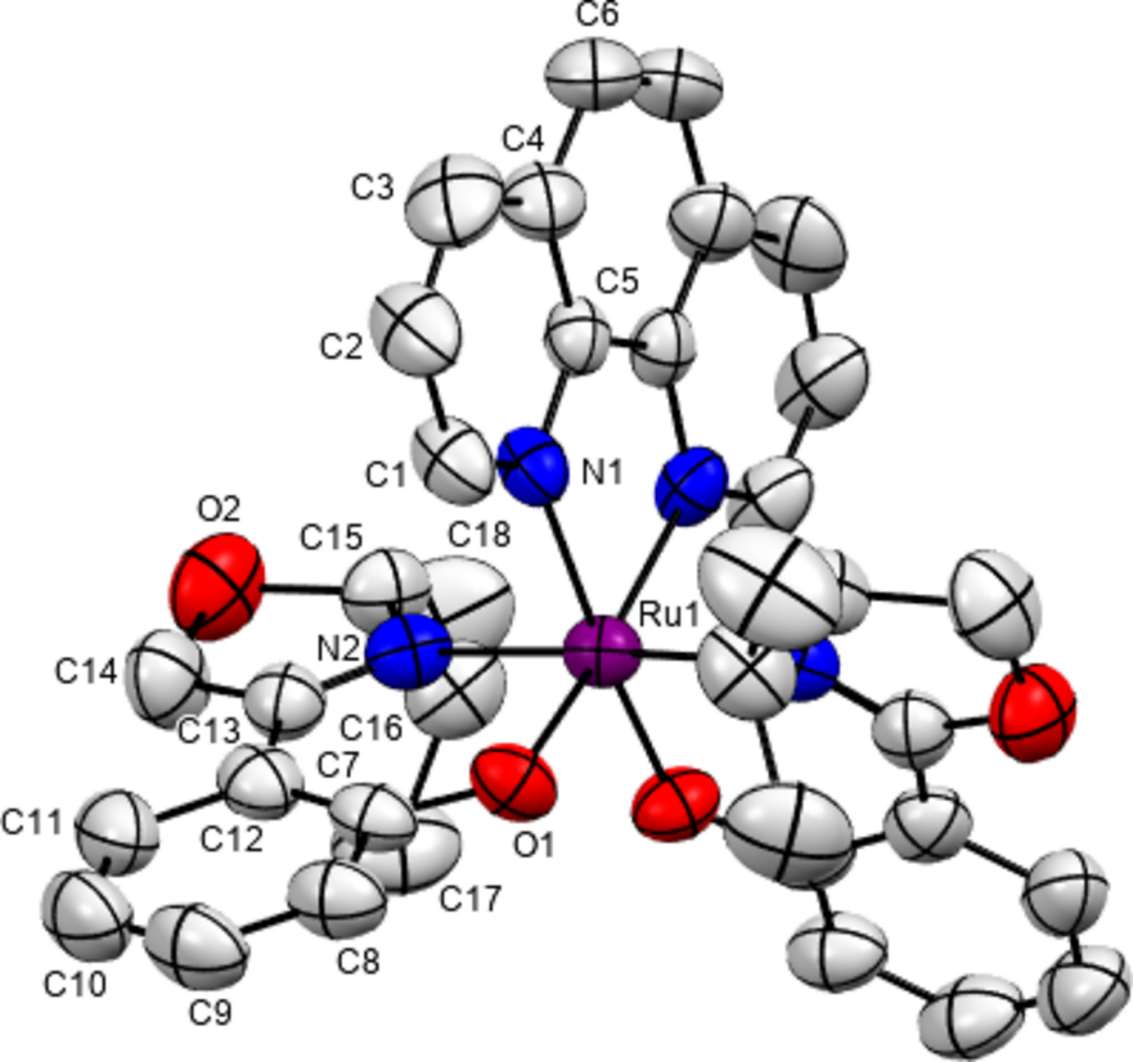
The mol­ecular structure of the title compound drawn with displacement ellipsoids at the 50% probability level; hydrogen atoms and the PF_6_^−^ counter-anion were removed for clarity. Non-labelled atoms are generated by a twofold rotation axis (symmetry operation: *y*, *x*, –*z*).

**Figure 2:**
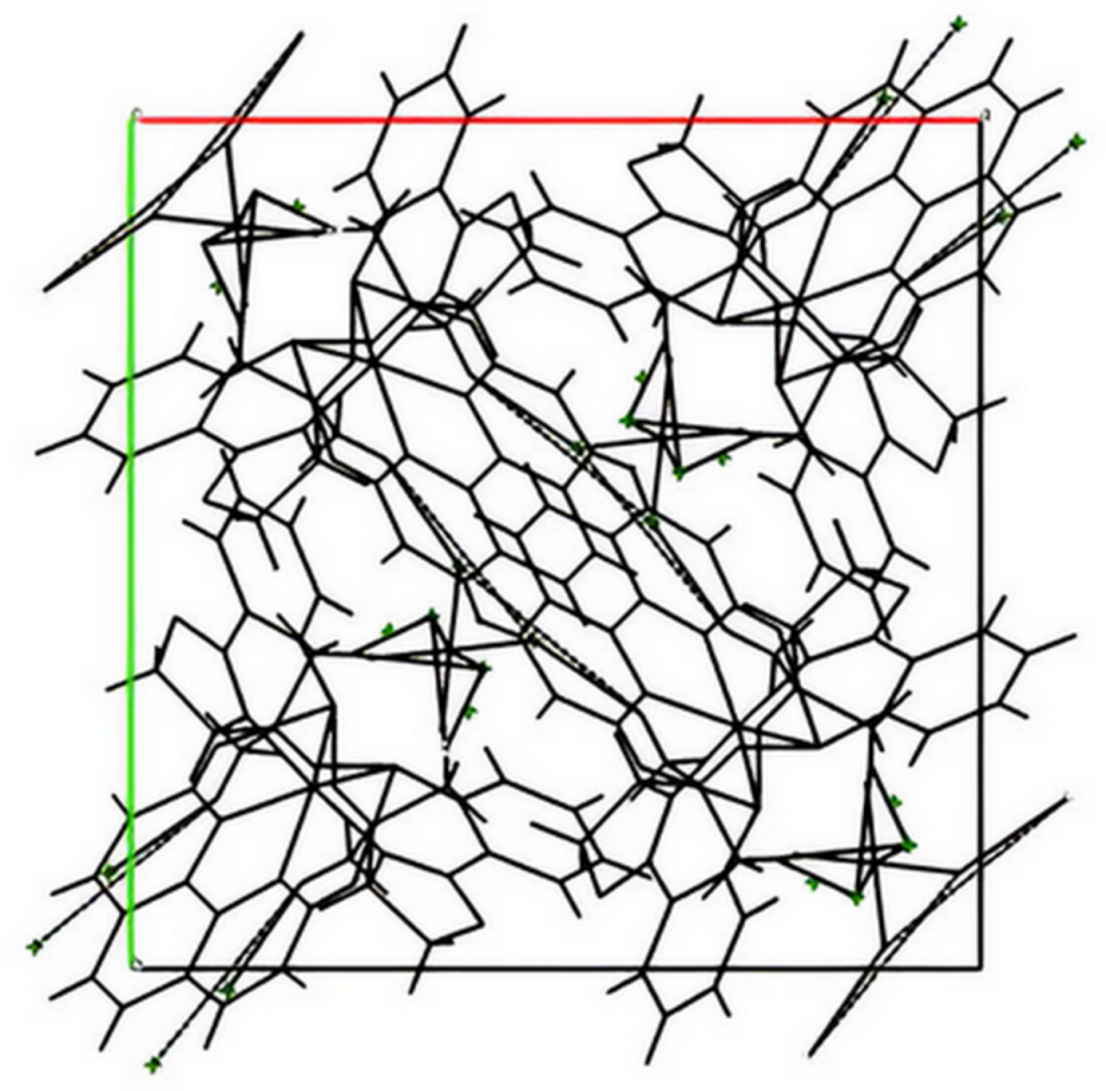
Crystal packing arrangement of the title compound in a view along the *c* axis. Non-classical hydrogen-bonding inter­actions are indicated by dotted lines.

The complex cation of the title salt constitutes of two optically pure bidentate salicyloxazoline ligands and a phenanthroline co-ligand arranged within an octa­hedral coordination sphere around the central Ru^III^ atom, which is located about a twofold rotation axis bis­ecting the phenantroline ligand (Fig. 1 ▸). This right-handed twist of the ligands leads to a Δ stereochemical configuration of the complex; the correctness of the absolute configuration is indicated by a Flack parameter (Parsons *et al.*, 2013 ▸) value of −0.003 (14). The bite angles, 89.76 (15)°, for the salicyloxazoline ligands are comparable with reported values, *e.g.* 86.68° (Brunner *et al.*, 1998 ▸), 88.29° (Davenport *et al.*, 2004 ▸), 86.88° (Kelani *et al.*, 2024 ▸), or 90.00 (Gong *et al.*, 2010 ▸) while that for the phenanthroline ligand, 79.0 (2)°, is almost similar to that of 80.12° (Gong *et al.*, 2010 ▸). The bond lengths of the Ru^III^ atom with the ligating atoms of 1.974 (3), 2.079 (4) and 2.072 (4) Å to O1, N1(phenanthroline) and N2(salicyloxazoline) atoms, respectively, also agree well with reported values. The crystal packing (Fig. 2 ▸) includes the disordered PF_6_^−^ counter-anion (located about a twofold rotation axis). Non-classical inter­molecular inter­actions featuring C—H⋯O and C—H⋯F contacts (Table 1 ▸) are present.

## Synthesis and crystallization

Di­chlorido-bis­(1,10-phenanthroline)ruthenium(II) (50.0 mg, 0.09 mmol, 1 eq) was added to (*S*)-isopropyl-2-(2-hy­droxy­phen­yl)oxazoline (38.5 mg, 0.2 mmol, 2 eq) in ethanol in the presence of K_2_CO_3_ (26.0 mg, 0.2 mmol, 2 eq). The reaction mixture was refluxed for 6 h under continuous stirring after which it was cooled to room temperature and then concentrated *in vacuo* under reduced pressure. The crude product was purified by column chromatography with silica gel using a solvent system of CH_2_Cl_2_:CH_3_OH:CH_3_CN = 9.7:0.2:0.1 *v*:*v*:*v*) to obtain a purple crystalline compound. Yield, 31 mg (46%, 0.04 mmol).

## Refinement

Details of the data collection, solution and refinement are given in Table 2 ▸. The disordered PF_6_^−^ anion was treated as equally disordered around the twofold rotation axis and was kept stable with SADI, SIMU and DELU restraints in *SHELXL* (Sheldrick, 2015*b* ▸). The highest remaining maximum and minimum electron density are 1.32 and 0.76 Å away from F1*A* and Ru1, respectively.

## Supplementary Material

Crystal structure: contains datablock(s) I. DOI: 10.1107/S2414314624008939/wm4221sup1.cif

Structure factors: contains datablock(s) I. DOI: 10.1107/S2414314624008939/wm4221Isup3.hkl

CCDC reference: 2383614

Additional supporting information:  crystallographic information; 3D view; checkCIF report

## Figures and Tables

**Table 1:** Hydrogen-bond geometry (Å, °)

*D*—H⋯*A*	*D*—H	H⋯*A*	*D*⋯*A*	*D*—H⋯*A*
C1—H1⋯O1^i^	0.95	2.59	3.102 (6)	114
C16—H16⋯O1^i^	1.00	2.53	3.224 (6)	126
C17—H17*A*⋯F3^ii^	0.98	2.52	3.464 (11)	162
C18—H18*A*⋯F2	0.98	2.48	3.357 (12)	149

Symmetry codes: (i) 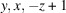; (ii) 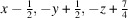.

**Table 2:** Experimental details

Crystal data	
Chemical formula	[Ru(C_12_H_14_NO_2_)_2_(C_12_H_8_N_2_)]PF_6_
*M*_r_	834.73
Crystal system, space group	Tetragonal, *P*4_1_2_1_2
Temperature (K)	173
*a*, *c* (Å)	15.3094 (13), 15.315 (2)
*V* (Å^3^)	3589.5 (8)
*Z*	4
Radiation type	Mo *K*α
μ (mm^−1^)	0.56
Crystal size (mm)	0.46 × 0.43 × 0.42

Data collection	
Diffractometer	Bruker APEXII CCD
Absorption correction	Multi-scan (*SADABS*; Krause *et al.*, 2015 ▸)
*T*_min_, *T*_max_	0.638, 0.746
No. of measured, independent and observed [*I* > 2σ(*I*)] reflections	53896, 4516, 3564
*R*_int_	0.067
(sin θ/λ)_max_ (Å^−1^)	0.669

Refinement	
*R*[*F*^2^ > 2σ(*F*^2^)], *wR*(*F*^2^), *S*	0.038, 0.107, 1.04
No. of reflections	4516
No. of parameters	245
No. of restraints	26
H-atom treatment	H-atom parameters constrained
Δρ_max_, Δρ_min_ (e Å^−3^)	0.38, −0.41
Absolute structure	Flack *x* determined using 1296 quotients [(*I*^+^)−(*I*^−^)]/[(*I*^+^)+(*I*^−^)] (Parsons *et al.*, 2013 ▸)
Absolute structure parameter	−0.003 (14)

Computer programs: *APEX2* and *SAINT* (Bruker, 2010 ▸), *SHELXT* (Sheldrick, 2015*a* ▸), *SHELXL* (Sheldrick, 2015*b* ▸), *Mercury* (Macrae *et al.*., 2020 ▸), *publCIF* (Westrip, 2010 ▸) and *WinGX* (Farrugia, 2012 ▸).

## full crystallographic data

### Δ-Bis[(S)-2-(4-isopropyl-4,5-dihydrooxazol-2-yl)phenolato-κ2N,O1](1,10-phenanthroline-κ2N,N')ruthenium(III) hexafluoridophosphate Crystal data

**Table:**

[Ru(C_12_H_14_NO_2_)_2_(C_12_H_8_N_2_)]PF_6_	*D*_x_ = 1.545 Mg m^−^^3^
*M**_r_* = 834.73	Mo *K*α radiation, λ = 0.71073 Å
Tetragonal, *P*4_1_2_1_2	Cell parameters from 8341 reflections
*a* = 15.3094 (13) Å	θ = 2.3–20.4°
*c* = 15.315 (2) Å	µ = 0.56 mm^−^^1^
*V* = 3589.5 (8) Å^3^	*T* = 173 K
*Z* = 4	Cuboid, purple
*F*(000) = 1700	0.46 × 0.43 × 0.42 mm

### Δ-Bis[(S)-2-(4-isopropyl-4,5-dihydrooxazol-2-yl)phenolato-κ2N,O1](1,10-phenanthroline-κ2N,N')ruthenium(III) hexafluoridophosphate Data collection

**Table:**

Bruker APEXII CCD diffractometer	4516 independent reflections
Radiation source: sealed-tube	3564 reflections with *I* > 2σ(*I*)
Triumph monochromator	*R*_int_ = 0.067
φ and ω scans	θ_max_ = 28.4°, θ_min_ = 1.9°
Absorption correction: multi-scan (*SADABS*; Krause *et al.*, 2015)	*h* = −20→20
*T*_min_ = 0.638, *T*_max_ = 0.746	*k* = −20→20
53896 measured reflections	*l* = −20→20

### Δ-Bis[(S)-2-(4-isopropyl-4,5-dihydrooxazol-2-yl)phenolato-κ2N,O1](1,10-phenanthroline-κ2N,N')ruthenium(III) hexafluoridophosphate Refinement

**Table:**

Refinement on *F*^2^	Secondary atom site location: difference Fourier map
Least-squares matrix: full	Hydrogen site location: inferred from neighbouring sites
*R*[*F*^2^ > 2σ(*F*^2^)] = 0.038	H-atom parameters constrained
*wR*(*F*^2^) = 0.107	*w* = 1/[σ^2^(*F*_o_^2^) + (0.0587*P*)^2^ + 0.7144*P*] where *P* = (*F*_o_^2^ + 2*F*_c_^2^)/3
*S* = 1.04	(Δ/σ)_max_ < 0.001
4516 reflections	Δρ_max_ = 0.38 e Å^−^^3^
245 parameters	Δρ_min_ = −0.41 e Å^−^^3^
26 restraints	Absolute structure: Flack *x* determined using 1296 quotients [(*I*^+^)-(*I*^-^)]/[(*I*^+^)+(*I*^-^)] (Parsons *et al.*, 2013)
Primary atom site location: structure-invariant direct methods	Absolute structure parameter: −0.003 (14)

### Δ-Bis[(S)-2-(4-isopropyl-4,5-dihydrooxazol-2-yl)phenolato-κ2N,O1](1,10-phenanthroline-κ2N,N')ruthenium(III) hexafluoridophosphate Special details

**Table:**

Geometry. All esds (except the esd in the dihedral angle between two l.s. planes) are estimated using the full covariance matrix. The cell esds are taken into account individually in the estimation of esds in distances, angles and torsion angles; correlations between esds in cell parameters are only used when they are defined by crystal symmetry. An approximate (isotropic) treatment of cell esds is used for estimating esds involving l.s. planes.

### Δ-Bis[(S)-2-(4-isopropyl-4,5-dihydrooxazol-2-yl)phenolato-κ2N,O1](1,10-phenanthroline-κ2N,N')ruthenium(III) hexafluoridophosphate Fractional atomic coordinates and isotropic or equivalent isotropic displacement parameters (Å^2^)

**Table:**

	*x*	*y*	*z*	*U*_iso_*/*U*_eq_	Occ. (<1)
C1	0.2882 (4)	0.4278 (4)	0.6429 (3)	0.0615 (13)	
H1	0.237967	0.400278	0.666771	0.074*	
C2	0.3215 (4)	0.5005 (4)	0.6832 (4)	0.0760 (18)	
H2	0.294934	0.521584	0.735125	0.091*	
C3	0.3917 (5)	0.5426 (4)	0.6499 (4)	0.0828 (19)	
H3	0.412522	0.594417	0.676826	0.099*	
C4	0.4345 (4)	0.5090 (4)	0.5742 (4)	0.0615 (13)	
C5	0.3979 (3)	0.4353 (3)	0.5381 (3)	0.0463 (10)	
C6	0.5100 (4)	0.5455 (4)	0.5356 (4)	0.0723 (16)	
H6	0.535851	0.595990	0.560765	0.087*	
C7	0.3015 (3)	0.1129 (3)	0.4108 (3)	0.0539 (12)	
C8	0.2786 (4)	0.0607 (4)	0.3381 (3)	0.0678 (14)	
H8	0.238849	0.082913	0.295931	0.081*	
C9	0.3130 (5)	−0.0217 (4)	0.3273 (4)	0.0815 (19)	
H9	0.295857	−0.055716	0.278301	0.098*	
C10	0.3710 (5)	−0.0552 (4)	0.3853 (5)	0.0852 (19)	
H10	0.393287	−0.112595	0.377536	0.102*	
C11	0.3975 (4)	−0.0054 (4)	0.4556 (5)	0.0759 (17)	
H11	0.439237	−0.028411	0.495301	0.091*	
C12	0.3633 (4)	0.0795 (3)	0.4695 (3)	0.0592 (12)	
C13	0.3904 (3)	0.1261 (4)	0.5476 (3)	0.0553 (12)	
C14	0.4704 (4)	0.1478 (5)	0.6678 (4)	0.0838 (19)	
H14A	0.529714	0.171713	0.658200	0.101*	
H14B	0.468526	0.119533	0.725909	0.101*	
C15	0.4011 (3)	0.2208 (4)	0.6615 (3)	0.0618 (13)	
H15	0.430056	0.279348	0.662139	0.074*	
C16	0.3308 (4)	0.2163 (4)	0.7321 (3)	0.0730 (15)	
H16	0.285475	0.260943	0.717068	0.088*	
C17	0.2847 (5)	0.1280 (5)	0.7371 (4)	0.102 (2)	
H17A	0.240548	0.129560	0.783417	0.152*	
H17B	0.256291	0.115575	0.681064	0.152*	
H17C	0.327529	0.082258	0.749904	0.152*	
C18	0.3698 (6)	0.2424 (7)	0.8209 (4)	0.122 (3)	
H18A	0.398851	0.299210	0.815570	0.183*	
H18B	0.323026	0.246367	0.864392	0.183*	
H18C	0.412379	0.198299	0.839249	0.183*	
N1	0.3248 (2)	0.3946 (3)	0.5709 (2)	0.0483 (9)	
N2	0.3638 (3)	0.2026 (3)	0.5728 (2)	0.0523 (9)	
O1	0.2614 (2)	0.1896 (2)	0.4175 (2)	0.0570 (9)	
O2	0.4479 (3)	0.0854 (3)	0.5994 (3)	0.0767 (11)	
F1A	0.6349 (16)	0.2780 (8)	0.7374 (14)	0.203 (8)	0.5
F1B	0.6026 (17)	0.3041 (13)	0.8114 (17)	0.250 (11)	0.5
F2	0.5257 (5)	0.3866 (6)	0.7660 (8)	0.247 (5)	
F3	0.6459 (7)	0.4151 (7)	0.8395 (6)	0.241 (4)	
P1	0.6246 (2)	0.3754 (2)	0.750000	0.1405 (16)	
Ru1	0.28566 (2)	0.28566 (2)	0.500000	0.04461 (15)	

### Δ-Bis[(S)-2-(4-isopropyl-4,5-dihydrooxazol-2-yl)phenolato-κ2N,O1](1,10-phenanthroline-κ2N,N')ruthenium(III) hexafluoridophosphate Atomic displacement parameters (Å^2^)

**Table:**

	*U*^11^	*U*^22^	*U*^33^	*U*^12^	*U*^13^	*U*^23^
C1	0.049 (3)	0.084 (4)	0.051 (2)	0.001 (3)	0.006 (2)	−0.025 (2)
C2	0.064 (3)	0.093 (4)	0.072 (3)	0.001 (3)	0.007 (3)	−0.046 (3)
C3	0.096 (5)	0.072 (4)	0.080 (4)	0.000 (3)	−0.015 (4)	−0.036 (3)
C4	0.060 (3)	0.062 (3)	0.063 (3)	−0.004 (2)	−0.009 (3)	−0.013 (3)
C5	0.046 (2)	0.048 (2)	0.046 (2)	0.0082 (19)	−0.0020 (19)	−0.0070 (19)
C6	0.067 (4)	0.060 (3)	0.090 (4)	−0.017 (3)	−0.005 (3)	−0.006 (3)
C7	0.063 (3)	0.050 (3)	0.049 (2)	−0.013 (2)	0.009 (2)	−0.0008 (19)
C8	0.076 (4)	0.068 (3)	0.060 (3)	−0.015 (3)	0.009 (3)	−0.015 (3)
C9	0.098 (5)	0.065 (4)	0.081 (4)	−0.015 (3)	0.020 (4)	−0.025 (3)
C10	0.091 (5)	0.059 (4)	0.106 (5)	−0.001 (3)	0.021 (4)	−0.018 (4)
C11	0.069 (4)	0.060 (3)	0.099 (4)	0.001 (3)	0.020 (3)	−0.003 (3)
C12	0.063 (3)	0.056 (3)	0.060 (3)	−0.006 (2)	0.013 (2)	0.005 (2)
C13	0.055 (3)	0.057 (3)	0.054 (3)	−0.001 (2)	0.004 (2)	0.004 (2)
C14	0.078 (4)	0.108 (5)	0.066 (3)	0.011 (4)	−0.018 (3)	0.017 (4)
C15	0.063 (3)	0.073 (3)	0.049 (2)	−0.003 (3)	−0.012 (2)	0.005 (3)
C16	0.076 (3)	0.098 (4)	0.044 (3)	−0.001 (4)	−0.003 (2)	0.001 (3)
C17	0.110 (5)	0.124 (6)	0.071 (4)	−0.029 (5)	0.013 (4)	0.019 (4)
C18	0.125 (6)	0.195 (10)	0.047 (3)	−0.025 (6)	−0.005 (4)	−0.020 (4)
N1	0.042 (2)	0.061 (2)	0.0424 (18)	0.0039 (17)	−0.0001 (16)	−0.0117 (17)
N2	0.054 (2)	0.064 (3)	0.0390 (16)	−0.0075 (19)	−0.0008 (16)	0.0044 (19)
O1	0.071 (2)	0.0503 (19)	0.0500 (17)	−0.0074 (16)	−0.0063 (16)	−0.0046 (14)
O2	0.082 (3)	0.072 (3)	0.075 (3)	0.009 (2)	−0.015 (2)	0.012 (2)
F1A	0.29 (2)	0.091 (7)	0.225 (17)	0.020 (10)	−0.067 (18)	−0.003 (11)
F1B	0.24 (2)	0.145 (16)	0.36 (3)	−0.025 (16)	0.01 (2)	0.080 (16)
F2	0.149 (6)	0.235 (9)	0.357 (14)	−0.025 (6)	−0.022 (8)	−0.047 (11)
F3	0.268 (10)	0.262 (11)	0.194 (8)	−0.019 (9)	−0.078 (8)	−0.046 (8)
P1	0.1223 (17)	0.1223 (17)	0.177 (4)	−0.025 (2)	−0.051 (2)	−0.051 (2)
Ru1	0.04954 (19)	0.04954 (19)	0.0347 (2)	−0.0046 (2)	0.00213 (16)	−0.00213 (16)

### Δ-Bis[(S)-2-(4-isopropyl-4,5-dihydrooxazol-2-yl)phenolato-κ2N,O1](1,10-phenanthroline-κ2N,N')ruthenium(III) hexafluoridophosphate Geometric parameters (Å, º)

**Table:**

C1—N1	1.337 (5)	C13—N2	1.299 (7)
C1—C2	1.370 (8)	C13—O2	1.338 (6)
C1—H1	0.9500	C14—O2	1.458 (7)
C2—C3	1.353 (9)	C14—C15	1.544 (8)
C2—H2	0.9500	C14—H14A	0.9900
C3—C4	1.426 (8)	C14—H14B	0.9900
C3—H3	0.9500	C15—N2	1.499 (6)
C4—C5	1.375 (7)	C15—C16	1.528 (7)
C4—C6	1.414 (8)	C15—H15	1.0000
C5—N1	1.376 (6)	C16—C17	1.527 (10)
C5—C5^i^	1.421 (8)	C16—C18	1.538 (8)
C6—C6^i^	1.335 (12)	C16—H16	1.0000
C6—H6	0.9500	C17—H17A	0.9800
C7—O1	1.329 (6)	C17—H17B	0.9800
C7—C12	1.402 (7)	C17—H17C	0.9800
C7—C8	1.414 (7)	C18—H18A	0.9800
C8—C9	1.377 (9)	C18—H18B	0.9800
C8—H8	0.9500	C18—H18C	0.9800
C9—C10	1.356 (10)	N1—Ru1	2.079 (4)
C9—H9	0.9500	N2—Ru1	2.072 (4)
C10—C11	1.380 (9)	O1—Ru1	1.974 (3)
C10—H10	0.9500	F1A—P1	1.512 (12)
C11—C12	1.418 (8)	F1B—P1	1.479 (14)
C11—H11	0.9500	F2—P1	1.544 (8)
C12—C13	1.453 (7)	F3—P1	1.535 (8)
			
N1—C1—C2	121.6 (5)	H17A—C17—H17B	109.5
N1—C1—H1	119.2	C16—C17—H17C	109.5
C2—C1—H1	119.2	H17A—C17—H17C	109.5
C3—C2—C1	120.8 (5)	H17B—C17—H17C	109.5
C3—C2—H2	119.6	C16—C18—H18A	109.5
C1—C2—H2	119.6	C16—C18—H18B	109.5
C2—C3—C4	120.0 (5)	H18A—C18—H18B	109.5
C2—C3—H3	120.0	C16—C18—H18C	109.5
C4—C3—H3	120.0	H18A—C18—H18C	109.5
C5—C4—C6	119.3 (5)	H18B—C18—H18C	109.5
C5—C4—C3	115.8 (5)	C1—N1—C5	118.0 (4)
C6—C4—C3	124.9 (5)	C1—N1—Ru1	127.9 (4)
C4—C5—N1	123.8 (4)	C5—N1—Ru1	114.1 (3)
C4—C5—C5^i^	119.8 (3)	C13—N2—C15	108.5 (4)
N1—C5—C5^i^	116.4 (2)	C13—N2—Ru1	125.1 (3)
C6^i^—C6—C4	120.9 (3)	C15—N2—Ru1	126.4 (4)
C6^i^—C6—H6	119.5	C7—O1—Ru1	128.4 (3)
C4—C6—H6	119.5	C13—O2—C14	106.1 (4)
O1—C7—C12	125.7 (4)	F1B—P1—F1B^ii^	138 (2)
O1—C7—C8	116.5 (5)	F1B—P1—F1A^ii^	93.8 (14)
C12—C7—C8	117.8 (5)	F1B^ii^—P1—F1A^ii^	51.5 (10)
C9—C8—C7	121.1 (6)	F1A—P1—F1A^ii^	79.1 (18)
C9—C8—H8	119.4	F1B—P1—F3	76.9 (11)
C7—C8—H8	119.4	F1B^ii^—P1—F3	108.7 (11)
C10—C9—C8	121.2 (6)	F1A—P1—F3	118.9 (9)
C10—C9—H9	119.4	F1A^ii^—P1—F3	73.6 (8)
C8—C9—H9	119.4	F1B—P1—F3^ii^	108.7 (11)
C9—C10—C11	119.6 (6)	F1B^ii^—P1—F3^ii^	76.9 (11)
C9—C10—H10	120.2	F1A—P1—F3^ii^	73.6 (8)
C11—C10—H10	120.2	F1A^ii^—P1—F3^ii^	118.9 (9)
C10—C11—C12	121.0 (7)	F3—P1—F3^ii^	165.1 (9)
C10—C11—H11	119.5	F1B—P1—F2	75.9 (10)
C12—C11—H11	119.5	F1B^ii^—P1—F2	143.1 (12)
C7—C12—C11	119.1 (5)	F1A—P1—F2	103.4 (10)
C7—C12—C13	122.9 (5)	F1A^ii^—P1—F2	163.5 (9)
C11—C12—C13	117.9 (5)	F3—P1—F2	91.3 (7)
N2—C13—O2	116.7 (4)	F3^ii^—P1—F2	77.1 (5)
N2—C13—C12	126.7 (5)	F1B—P1—F2^ii^	143.1 (12)
O2—C13—C12	116.6 (5)	F1B^ii^—P1—F2^ii^	75.9 (10)
O2—C14—C15	105.4 (4)	F1A—P1—F2^ii^	163.5 (9)
O2—C14—H14A	110.7	F1A^ii^—P1—F2^ii^	103.4 (10)
C15—C14—H14A	110.7	F3—P1—F2^ii^	77.1 (5)
O2—C14—H14B	110.7	F3^ii^—P1—F2^ii^	91.3 (7)
C15—C14—H14B	110.7	F2—P1—F2^ii^	78.9 (8)
H14A—C14—H14B	108.8	O1—Ru1—O1^i^	97.4 (2)
N2—C15—C16	111.3 (4)	O1—Ru1—N2	89.76 (15)
N2—C15—C14	100.6 (4)	O1^i^—Ru1—N2	88.30 (15)
C16—C15—C14	114.1 (5)	O1—Ru1—N2^i^	88.30 (15)
N2—C15—H15	110.2	O1^i^—Ru1—N2^i^	89.77 (15)
C16—C15—H15	110.2	N2—Ru1—N2^i^	177.1 (2)
C14—C15—H15	110.2	O1—Ru1—N1	170.53 (15)
C17—C16—C15	113.6 (5)	O1^i^—Ru1—N1	91.80 (15)
C17—C16—C18	111.4 (5)	N2—Ru1—N1	92.57 (16)
C15—C16—C18	109.9 (5)	N2^i^—Ru1—N1	89.70 (15)
C17—C16—H16	107.2	O1—Ru1—N1^i^	91.80 (15)
C15—C16—H16	107.2	O1^i^—Ru1—N1^i^	170.53 (15)
C18—C16—H16	107.2	N2—Ru1—N1^i^	89.70 (15)
C16—C17—H17A	109.5	N2^i^—Ru1—N1^i^	92.56 (16)
C16—C17—H17B	109.5	N1—Ru1—N1^i^	79.0 (2)
			
N1—C1—C2—C3	1.6 (10)	O2—C14—C15—N2	−15.9 (6)
C1—C2—C3—C4	−2.9 (11)	O2—C14—C15—C16	103.4 (5)
C2—C3—C4—C5	2.0 (9)	N2—C15—C16—C17	58.9 (7)
C2—C3—C4—C6	−177.6 (7)	C14—C15—C16—C17	−54.2 (7)
C6—C4—C5—N1	179.7 (5)	N2—C15—C16—C18	−175.5 (6)
C3—C4—C5—N1	0.1 (8)	C14—C15—C16—C18	71.4 (8)
C6—C4—C5—C5^i^	−1.3 (9)	C2—C1—N1—C5	0.5 (8)
C3—C4—C5—C5^i^	179.1 (6)	C2—C1—N1—Ru1	179.8 (5)
C5—C4—C6—C6^i^	0.8 (11)	C4—C5—N1—C1	−1.3 (7)
C3—C4—C6—C6^i^	−179.6 (7)	C5^i^—C5—N1—C1	179.6 (5)
O1—C7—C8—C9	−176.8 (5)	C4—C5—N1—Ru1	179.2 (4)
C12—C7—C8—C9	2.8 (8)	C5^i^—C5—N1—Ru1	0.2 (6)
C7—C8—C9—C10	−1.0 (10)	O2—C13—N2—C15	−5.2 (6)
C8—C9—C10—C11	−1.2 (10)	C12—C13—N2—C15	173.8 (5)
C9—C10—C11—C12	1.6 (10)	O2—C13—N2—Ru1	173.3 (3)
O1—C7—C12—C11	177.1 (5)	C12—C13—N2—Ru1	−7.7 (7)
C8—C7—C12—C11	−2.4 (7)	C16—C15—N2—C13	−108.2 (5)
O1—C7—C12—C13	1.0 (8)	C14—C15—N2—C13	13.0 (6)
C8—C7—C12—C13	−178.5 (5)	C16—C15—N2—Ru1	73.3 (6)
C10—C11—C12—C7	0.3 (8)	C14—C15—N2—Ru1	−165.4 (4)
C10—C11—C12—C13	176.5 (5)	C12—C7—O1—Ru1	8.7 (7)
C7—C12—C13—N2	−1.2 (8)	C8—C7—O1—Ru1	−171.7 (3)
C11—C12—C13—N2	−177.3 (5)	N2—C13—O2—C14	−5.9 (6)
C7—C12—C13—O2	177.9 (5)	C12—C13—O2—C14	175.0 (5)
C11—C12—C13—O2	1.7 (7)	C15—C14—O2—C13	13.9 (6)

Symmetry codes: (i) *y*, *x*, −*z*+1; (ii) −*y*+1, −*x*+1, −*z*+3/2.

### Δ-Bis[(S)-2-(4-isopropyl-4,5-dihydrooxazol-2-yl)phenolato-κ2N,O1](1,10-phenanthroline-κ2N,N')ruthenium(III) hexafluoridophosphate Hydrogen-bond geometry (Å, º)

**Table:**

*D*—H···*A*	*D*—H	H···*A*	*D*···*A*	*D*—H···*A*
C1—H1···O1^i^	0.95	2.59	3.102 (6)	114
C16—H16···O1^i^	1.00	2.53	3.224 (6)	126
C17—H17*A*···F3^iii^	0.98	2.52	3.464 (11)	162
C18—H18*A*···F2	0.98	2.48	3.357 (12)	149

Symmetry codes: (i) *y*, *x*, −*z*+1; (iii) *x*−1/2, −*y*+1/2, −*z*+7/4.
